# Utility of cfDNA Fragmentation Patterns in Designing the Liquid Biopsy Profiling Panels to Improve Their Sensitivity

**DOI:** 10.3389/fgene.2019.00194

**Published:** 2019-03-12

**Authors:** Maxim Ivanov, Polina Chernenko, Valery Breder, Konstantin Laktionov, Ekaterina Rozhavskaya, Sergey Musienko, Ancha Baranova, Vladislav Mileyko

**Affiliations:** ^1^Department of Biological and Medical Physics, Moscow Institute of Physics and Technology, Dolgoprudny, Russia; ^2^N.N. Blokhin Russian Cancer Research Center, Moscow, Russia; ^3^Atlas Oncology Diagnostics, Ltd., Moscow, Russia; ^4^Vavilov Institute of General Genetics, Moscow, Russia; ^5^Research Centre for Medical Genetics, Moscow, Russia; ^6^School of Systems Biology, George Mason University, Fairfax, VA, United States

**Keywords:** NGS, cfDNA, liquid biopsy, cancer, DNA fragmentation, nucleosome, amplicon, primer design

## Abstract

Genotyping of cell-free DNA (cfDNA) in plasma samples has the potential to allow for a noninvasive assessment of tumor biology, avoiding the inherent shortcomings of tissue biopsy. Next generation sequencing (NGS), a leading technology for liquid biopsy analysis, continues to be hurdled with several major issues with cfDNA samples, including low cfDNA concentration and high fragmentation. In this study, by employing Ion Torrent PGM semiconductor technology, we performed a comparison between two multi-biomarker amplicon-based NGS panels characterized by a substantial difference in average amplicon length. In course of the analysis of the peripheral blood from 13 diagnostic non-small cell lung cancer patients, equivalence of two panels, in terms of overall diagnostic sensitivity and specificity was shown. A pairwise comparison of the allele frequencies for the same somatic variants obtained from the pairs of panel-specific amplicons, demonstrated an identical analytical sensitivity in range of 140 to 170 bp amplicons in size. Further regression analysis between amplicon length and its coverage, illustrated that NGS sequencing of plasma cfDNA equally tolerates amplicons with lengths in the range of 120 to 170 bp. To increase the sensitivity of mutation detection in cfDNA, we performed a computational analysis of the features associated with genome-wide nucleosome maps, evident from the data on the prevalence of cfDNA fragments of certain sizes and their fragmentation patterns. By leveraging the support vector machine-based machine learning approach, we showed that a combination of nucleosome map associated features with GC content, results in the increased accuracy of prediction of high inter-sample sequencing coverage variation (areas under the receiver operating curve: 0.75, 95% CI: 0.750–0.752 vs. 0.65, 95% CI: 0.63–0.67). Thus, nucleosome-guided fragmentation should be utilized as a guide to design amplicon-based NGS panels for the genotyping of cfDNA samples.

## Introduction

In an approach known as “liquid biopsy,” cell-free DNA (cfDNA) which circulates in the plasma may be used for a diagnostic detection of tumor-specific mutations (Dawson et al., 2013; Pupilli et al., 2013; Xi et al., 2016). In the frame of the Lab-Developed Tests (LDT) paradigm, analysis of cfDNA has already gained approval for a number of common indications, including the detection of the resistance mutation T790M in the EGFR encoding gene (Malapelle et al., 2016), which commonly emerges in lung adenocarcinomas treated with tyrosine kinase inhibitors.

At their inception, cfDNA-based LDTs commonly exploited one or another conventional DNA analysis technique, including real-time PCR, droplet digital PCR and beads, emulsions, amplification, and magnetics (BEAM)ing digital PCR (Dawson et al., 2013; Oxnard et al., 2014; Siravegna et al., 2015; Thress et al., 2015; Sacher et al., 2016). Many studies showed that the concordance of liquid biopsy and tissue-based analysis is relatively high; nevertheless, these approaches are not free of limitations. Typically, PCR-based and hybridization-based cfDNA profiling techniques are developed to detect particular DNA variants, which most commonly underlie one or another previously described pathophysiological process. These and other variant-specific techniques are not suitable for the exploratory analysis of cfDNA, which is necessary for acquisition of knowledge concerning non-conventional, emerging resistance pathways, for co-detection of the mismatch repair phenotype, and for off-label prescribing of anticancer medications commonly required for personalized treatment of metastatic tumors (Tafe et al., 2015; Wei et al., 2016; Zehir et al., 2017). These limitations are readily surmounted by an advent of sequencing-based technologies, including whole exome sequencing or, more applicable to cfDNA analysis, amplicon-based panels, which are limited to their target genes, but are still exploration-permissive.

With reported sensitivity and a specificity of more than 80%, and 98 to 100%, respectively (Krishnamurthy et al., 2017), a next generation sequencing (NGS) analysis of cfDNA has already inserted itself into the ranks of the commonly used LDTs. Nevertheless, further improvement of the sensitivity in liquid biopsy-based tests is warranted. The most common way to improve sensitivity of the mutation detection in liquid biopsy samples, is to increase the coverage, which in turn leads to a substantial increase in the cost of an assay. Deep or ultradeep coverage is necessary in order to account for low concentrations of total cfDNA in plasma samples that are compounded by the dilution of tumor-specific cfDNA fragments, by substantial amounts of non-tumoral cfDNA fragments (Hellwig et al., 2018).

Another physical characteristic of cfDNA, the distribution of the sizes of its fragments, is relevant to the detection of DNA variants both by sequencing and by PCR. Recent whole-genome sequencing (WGS) studies of cfDNA demonstrated that the distribution of the sizes of plasma derived DNA fragments is far from the typical lognormal distribution that reflects the patterning of DNA in formalin fixed-paraffin-embedded samples or snap-frozen tissues. In fact, cfDNA exhibits a predominant peak at a fragment length of ∼167 bp accompanied by the second, significantly less pronounced extremum at around 350 bp (Ma et al., 2017). These observations mean that the majority of these fragments are suitable to assess the technique that relies on conventional lengths of PCR amplicons. It is of note that tumor-derived cfDNA fragments are even shorter than those that originate from healthy cells of the same origin (Jiang et al., 2015). In the domain of conventional systems for the detection of DNA variants, these characteristic of cfDNA have prompted the development of ultra-short amplicon PCR, which allows for the substantial increase of analytical and, as a consequence, diagnostic sensitivity of these assays.

Moreover, recent studies have shown that fragmentation pattern of cfDNA is not random. As cfDNA degradation is guided by nucleosome patterns defined by epigenetic regulation within particular loci (Ivanov et al., 2015), recurrent underrepresentation of some regions in cfDNA introduces systematic bias in the PCR based enrichment of target amplicons and undermine the sensitivity at a local scale.

In this study, we investigated the effect of the amplicon length on the diagnostic and analytical sensitivity of mutation detection, using two amplicon-based NGS panels with diverse amplicon lengths. We also describe ways to utilize the knowledge of cfDNA fragmentation patterns to increase the sensitivity of mutation detection in a liquid biopsy setting.

## Materials and Methods

### Sample Collection

The sequencing was performed on cfDNA fragments extracted from previously collected plasma samples of 13 non-small cell lung cancer (NSCLC) patients, treated at the Blokhin Russian Cancer Research Centre in 2014 to 2015. For each patient, tumor tissue-based EGFR mutation status was assessed using the therascreen EGFR RGQ PCR Kit (Qiagen, Milan, Italy) according to the manufacturers protocol.

For nucleosome-guided cfDNA fragmentation pattern analysis we used publicly available, anonymized WGS data of cfDNA, described by Snyder et al. (2016) and included in dataset [PRJNA291063].

The present study was approved by the Atlas Biomed Internal Review Board. All subjects gave written informed consent in accordance with the Declaration of Helsinki.

### DNA Extraction and Sample Quality Control

For each NSCLC patient, a peripheral blood sample was collected into an EDTA-containing vacutainer tube (BD). Samples were fractionated into plasma and blood cells by centrifugation at 400 *g* for 15 min within 4 h after venipuncture, followed by a secondary spin at 1200 *g* for 20 min. Resultant plasma samples were frozen in aliquots and stored at -80°C until DNA isolation. Circulating DNA was extracted from 4 ml of plasma using the Blood Plasma DNA Isolation Kit (BioSilica Ltd., Russia) according to the manufacturer’s instructions, eluted by 120 μl of nuclease-free water, mixed with 3 μl of glycogen (20 mg/ml, Fermentas, Lithuania), 1/10 volume of 50 mM triethylamine and then precipitated with 5 volumes of acetone (Bryzgunova et al., 2011). After reconstitution in 30–50 μl of water, cfDNA concentrations were measured using the Qubit fluorometer.

### Library Preparation and Quality Control

Sequencing libraries were prepared according to the manufacturer’s protocol for Ion AmpliSeq Cancer Hotspot Panel (ITCHP2), designed to amplify 207 target regions across 50 cancer-related genes. Additionally, a custom panel namely Atlas Clinical Panel (AODCP), was designed to cover the following genes: EGFR, IDH2, NRAS, KIT, BRAF, TP53, PDGFRA, PTEN, IDH1, KRAS, PIK3CA, ERBB2, CTNNB1 (AODCP, 55 target regions). The custom panel was designed using the Ion AmpliSeq Designer server (pipeline version 5.2). The two panels had several loci in common, allowing for their comparison.

### Sequencing and Data Analysis

Pooled libraries were sequenced employing Ion Torrent PGM, according to the manufacturers protocol. As low frequency mutant alleles were expected, initial analysis was performed using Ion Torrent Suite software (version 5.2.0) on low stringency settings. In order to exclude false negative single nucleotide variant (SNV) calls, concomitant Bowtie2-Strelka pipeline analysis was carried out. After aligning all reads to the genome (GRCh37) (Bowtie2 parameters: –rdg 5,2 –rfg 5,2 -N 1 -L 17), further off-target reads were removed, while the remaining reads were realigned on target sequences. Primer sequences were excluded from reads employing in-house software (Ivanov et al., 2018). Somatic variant calling was performed employing Strelka (maxInputDepth set to -1; indelMaxRefRepeat set to 6; indelMaxWindowFilteredBasecallFrac set to 0.4; indelMaxIntHpolLength set to 6; lower quality bound for SNV and indels set to 9 and 2, respectively). Variants supported with less than 20 reads in total were discarded. If less than four reads supported alternative allele, the variant was omitted. Mutation hotspots were defined as nucleotide variations identified in ten or more COSMIC (Forbes et al., 2010) samples. Detected variants located within mutation hotspots were supposed to be confidently somatic. Variants outside mutation hotspots with minor allele frequency in the general population, as defined by 1000 Genomes Project (1000 Genomes Project Consortium et al., 2015), of 5% and more were supposed to be confidently germline. Further analysis was limited to confidently somatic and confidently germline variants. Preprocessed fastq files were additionally screened for mutation hotspots by inputting wild type and expected mutant reads into the Poisson distribution statistical model with complexity-dependent variable expectation probability of SNVs and indels. Somatic variant calls were verified manually, in the Tablet (version 1.16.09.06) read alignment visualization tool (Milne et al., 2010). Variant allele frequencies were quantified within raw read sets as a ratio of reads confirming the mutation to the total count of qualified reads covering the mutation site. Normalization of mutation allele frequencies to amplicon coverage was performed by bootstrapping. The genome variation analysis was limited to the nucleotide changes affecting the protein sequence, unless otherwise specified. Publicly available software and database versions used were Bowtie2 v. 2.1.0 (Langmead and Salzberg, 2012), Strelka v. 1.0.14 (Saunders et al., 2012), and SAMtools v. 0.1.19 (Li, 2011). COSMIC and dbSNP databases were assessed in December 2017.

GC content normalization for linear regression analysis was performed leveraging a simple adjustment according to the equation 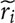 = *r_i_^m^/m*_GC_, where *r_i_* stands for the read count of the *i*th amplicon, *m_GC_* is the median read count of all windows with the same GC content as the *i*th amplicon, and *m* is the overall median of all the amplicons. Deviation of coverage from the mean was performed for 5% GC content bands rather than percentages of 0, 1, 2, 3, …, 100%. Linear regression analysis was performed employing simple least square fitting.

Nucleosome-guided cfDNA fragmentation patterns were analyzed in publicly available sequences obtained from plasma samples pooled from an unknown number of healthy individuals (GSM1833219). The details of the DNA extraction, library preparation and sequencing are provided in Snyder et al. (2016). Briefly, cfDNA libraries underwent paired-end sequencing with Illumina sequence-by-synthesis technology generating reads of 101 bp in size. Importantly, at the library preparation stage, plasma DNA samples did not undergo fragmentation by sonication and, thus, original cfDNA molecules were preserved, granting the opportunity to investigate its fragmentation patterns. The fastq read sequences were aligned to the human genome (aforementioned reference build) with BWA-mem v. 0.7.12 (Li and Durbin, 2009). cfDNA fragment length may exceed sequencing read length, however, paired-end sequencing allows to capture both start and end positions of the fragment. Paired reads, thus, continued to represent WGS fragments. Nucleosome position stringencies were calculated essentially as described in Valouev et al., using the NuMap software with standard parameters. NuMap performs the nucleosome mapping based on the kernel smoothed reads count calculation (Valouev et al., 2011).

For ITCHP2 and AODCP panel amplicons, fragment counts were generated *in silico* after matching both primers with the fragment amplified and sequenced experimentally. To understand the patterns of amplicon coverage by experimentally observed fragments, the fragments were generated using paired reads, then further filtered by length to include only fragments in the range of 80 to 250 bp. Dinucleosome fragments were therefore excluded. To improve resolution, resulting fragments were trimmed by 40 bp around dyads to generate a set of equal-length fragments. For each sequenced nucleotide position, counts of overlapping fragments were recorded. Generated data were subjected to a lowpass filter with the square pulse kernel with the width of 21 base pairs, then resulting coverage plots were mapped to amplicons genome positions.

Statistical analysis was performed using R, version 3.2.3. For machine learning, we used the open source library Orange (Demsar et al., 2013). Five machine learning algorithms were evaluated to find the best model, demonstrating the highest prediction accuracy based on all descriptors [support vector machine (SVM), neural network, multiple linear regression, naïve Bayes, and random forest].

## Results

### Sample Sequencing and Mutation Analysis

In this study, fourteen cfDNA samples collected from patients with NSCLC, were analyzed using the screening panels ITCHP2 and AODCP. The mean sequencing coverages across all experiments were set at 1150× for the AODCP panel and 802× for the ITCHP2 panel with corresponding medians of 1002× and 674×, respectively.

**FIGURE 1:**
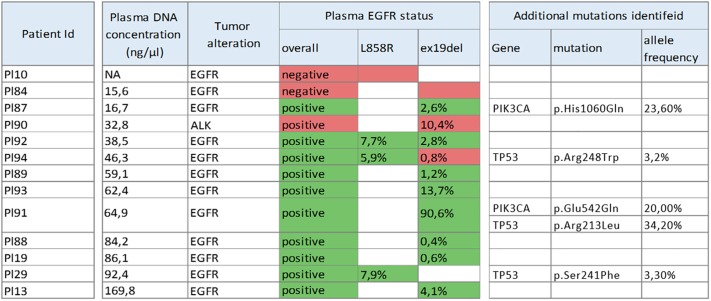
Samples used for data analysis as well as mutations identified during NGS sequencing and allele frequencies thereof (plasma EGFR status). Mutations identified employing a conventional sequencing method indicated in the tumor alteration column while its match (green) or mismatch (red) with NGS results specified in plasma EGFR status column.

Variant detection results were completely concordant for two panels across 18 identified somatic mutations. Plasma variant detection results were concordant with baseline tissue analysis in 9 samples (69%). False negative samples were limited to the cases, characterized with low plasma DNA concentration (Figure 1). In addition to mutations identified by tissue analysis at baseline, namely, these in EGFR and RAS, the sequencing of 13 plasma cfDNA samples revealed five additional somatic missense mutations, including these in PIK3CA and TP53 genes (Figure 1).

### Significance of Amplicon Length for Mutation Detection Sensitivity and Specificity

**FIGURE 2:**
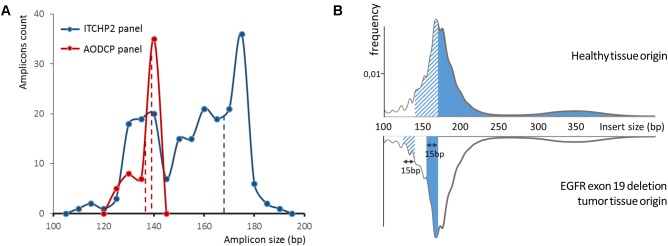
**(A)** Ion torrent cancer hotspot 2 (ITCHP2) and custom AODCP primer panels amplicon length distribution. A constant window of 5 bp was used to discretize amplicon length. Dotted lines demonstrate length of amplicons, covering exon 19 of the EGFR. **(B)** cfDNA fragment length distribution influence available for the amplification DNA molecules in plasma and, thus, amplification effectiveness. Solid fill at the top panel demonstrates the spectrum of cfDNA fragments involved in EGFR exon 19 PCR amplification employing the ITCHP2 panel. Dashed fill demonstrates the extension of that spectrum in case the AODCP panel is used. Fills in the bottom panel demonstrate the spectrum extension for two panels, respectively, in case of the 15 bp exon 19 deletion mutation.

The average length of amplicons in panel AODCP was much shorter than that in panel ITCHP2 (Figure 2A), with median amplicon lengths to include primer sequences at 137 and 156 bp, respectively. Despite the difference in amplicon sizes, variant calling results obtained for each panel were completely concordant, with a total of 51 either somatic or germline variants detected. Therefore, diagnostic sensitivity and specificity of these two detection systems were the same at the study power.

**FIGURE 3:**
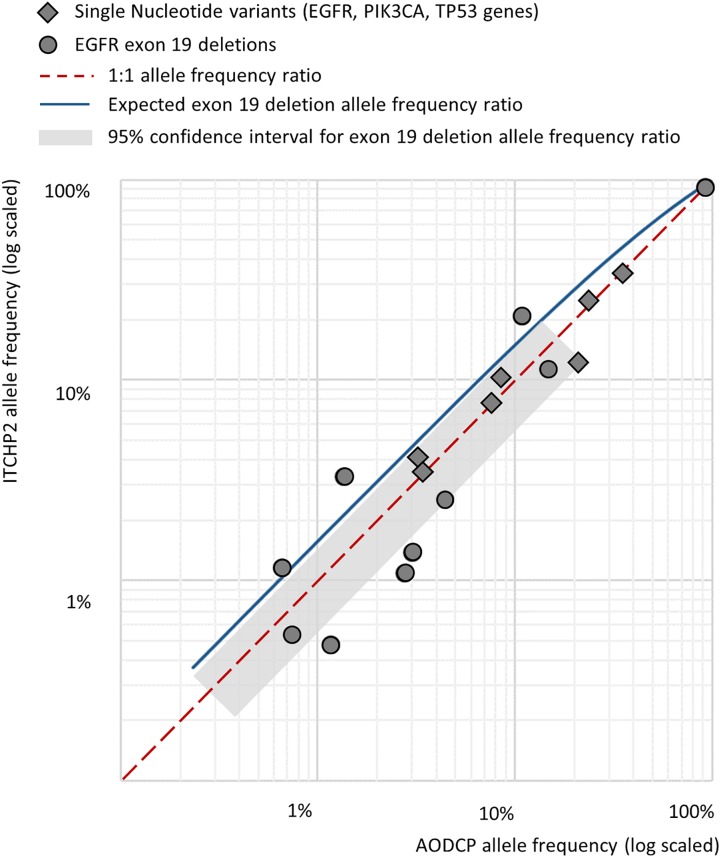
Pairwise comparison of the frequencies for same mutated allele in reads obtained from pairs of panel-specific amplicons across detected somatic variants.

In order to explore possible influences of the amplicon length on the limits of detection and, therefore, analytical sensitivity to the presence of the mutations in liquid biopsy, we performed a pairwise comparison of the frequencies for same mutated allele in reads obtained from pairs of panel-specific amplicons. For the synonymous germline variant, namely, EGFR p.Gln787= with the total of 15 alleles identified (1000 Genomes MAF 0.43), allele frequencies extracted from analysis of AODCP and ITCHP2 amplicons were equivalent (Wilcoxon signed rank test *p*-value = 0.88). On the other hand, analysis of somatic mutations, which are typically present in a relatively small fraction of the reads, showed Pearson’s correlation coefficients of 0.88 (*p*-value = 0.02; Wilcoxon signed rank test *p*-value = 0.44) for point mutations in genes *EGFR*, *TP53*, and *PIK3CA*, and 0.95 for the deletions of the *EGFR* exon 19 (*p*-value = 0.001; Wilcoxon signed rank test *p*-value = 0.53) (Figure 3). Since *EGFR* deletions further reduce the length of amplified fragments by 15 or more bp, their presence should, at least in theory, increase analytical sensitivity of the detection system (Figure 2B). Notably, the geometric mean ratio of the allele frequency of the EGFR exon 19 deletions, detected by two panels, was 1.16 (95% CI, 0.72–1.88; *p*-value > 0.1). This indicates that the analytical sensitivity of this assay is unlikely to change even if the difference in the average sizes of amplicons would increase further.

**FIGURE 4:**
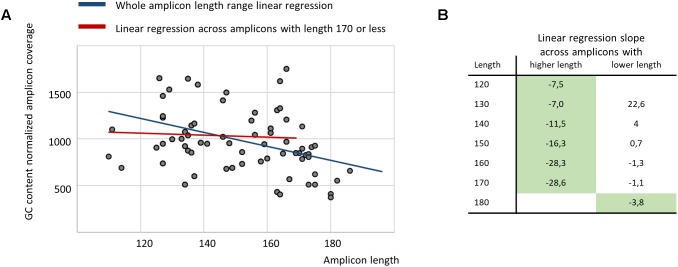
The regression analysis demonstrates no relationship between amplicon length and its average coverage when restricting the analysis to amplicons with a length of 170 or less **(A)**, while, broken stick regression indicates that the system suffers a shock after 170 bp **(B)** (green fill indicates statistically significant slope coefficient with *p*-value of 0.05 or less).

Finally, we performed a regression analysis to estimate the relationship between amplicon length and its average coverage across samples for the ITCHP2 panel, representing a wider spectrum of amplicon lengths. After normalization on GC-content and overall sample read count, linear regression analysis employing the least squares fitting approach, demonstrated a negative slope with a Student *t*-test *p*-value of 0.0063. However, regression analysis across the set of amplicons with a length of 170 bp or less yielded a non-significant slope coefficient (*p*-value 0.69) (Figure 4A,B). Regression analysis between amplicon length and its coverage covariance demonstrated no significant correlation in any amplicon length range (data not shown). Considering that amplicons with a length of 120 or less comprises of only 5% of that set, this indicates that the NGS sequencing of plasma cfDNA equally tolerates amplicons with a length in the 120–170 bp range.

### Nucleosome-Guided Pattern May Facilitate Primer Panel Design

**FIGURE 5:**
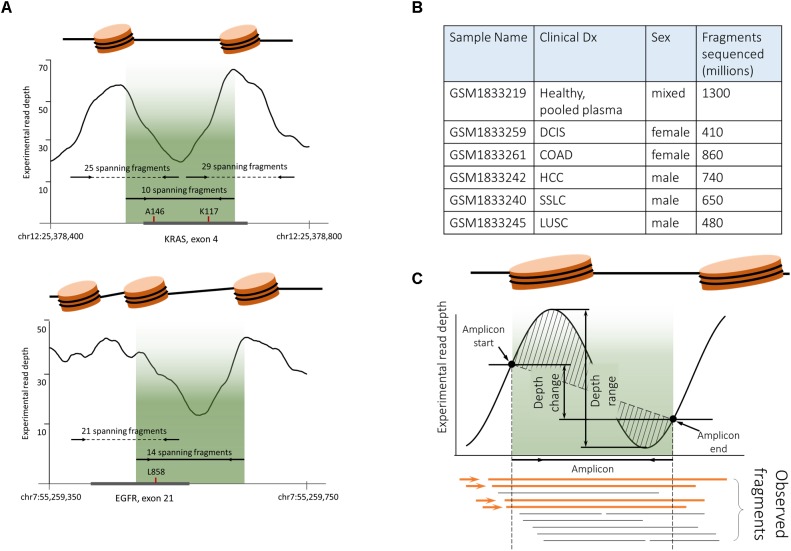
Plasma cfDNA fragmentation pattern biases analytical characteristics of PCR-based somatic detection system. **(A)** ITCHP2 primer panel design mapped to the nucleosome guided cfDNA fragmentation pattern indicates a possible bias in amplification effectiveness. Experimental coverage was assessed based on the pair-end WGS plasma sequencing of healthy individuals (GSM1833219). Fragment counts were calculated as WGS captured and sequenced fragments (continued read pairs), completely covering the amplicon of interest. Localization of the ITCHP2 panel amplicon (solid line with arrows, indicating primers as well as lightened area) in local minimum would result in the lack of availability for the amplification of cfDNA fragments. Shifting amplicons (dotted lines) within clinically relevant mutations may result in an increase of available fragments and thus amplification effectiveness. A146 and K117 indicates clinically relevant KRAS mutation hotspots in the exon of interest. A similar situation can be observed for the EGFR L858R mutation and respective amplicon design – moving amplicons within clinically relevant mutation site mutations may result in increased coverage uniformity. **(B)** Publicly available cfDNA WGS sequencing data [PRJNA291063] was used to generate cfDNA fragmentation maps. **(C)**. In order to decipher the complex variable of the cfDNA fragmentation pattern and its mapping to amplicons positions and lengths, four features were introduced, namely, observed fragments (Feature A), depth range (Feature B), depth change (Feature C), and depth shape (Feature D).

According to the most commonly cited hypothesis, plasma cfDNA originates from apoptotic cells where genomic DNA is digested by a set of nucleases (Ma et al., 2017). Wrapping around nucleosomes protects some of the DNA fragments from digestion; that is why cfDNA fragments correspond primarily to the mononucleosome bound regions. Originally supported only by a unimodal distribution of cfDNA fragments sizes (Fan et al., 2008; Lo et al., 2010), this hypothesis has been recently validated in several studies (Chandrananda et al., 2015; Snyder et al., 2016; Ulz et al., 2016). In particular, employing whole exome sequencing of cfDNA fragments to infer the read depth coverage allowed the construction of ‘plasma genome-wide nucleosome maps. Mapping the fragments covered by the ITCHP2 panel, to these nucleosome maps, showed that the positions of the ITCHP2 primers were selected in a non-optimal way with respect to the nucleosome positioning (*p*-value for nucleosome peaks and amplicons interception 0.36). An amplicon covering KRAS exon 4 serves as a good illustration for non-optimal selection of primers which fall in between two peaks (Figure 5A). Because of that, amounts of spanning cfDNA fragments are much lower than for the primers selected to amplify the fragment located within the same peak. A similar situation may be observed for the EGFR exon 21; shifting positions of the primers by the order of 100 nucleotides may result in an increase of the depth and the uniformity of the coverage, without compromising amplification of the clinically relevant, mutation-harboring locus.

At the next stage of analysis, we inquired whether efficiency of targeted resequencing of cfDNA samples depends on the pattern of DNA fragmentation. To perform this analysis, for all amplicons represented in the ITCHP2 panel, the fragmentation patterns were extracted from the repository of reads obtained after a shotgun sequencing of cfDNA fragments purified from the pool of plasma samples, of healthy individuals and from five individual patients with solid tumors (Figure 5B).

It is known that both the nucleosome positioning (Struhl and Segal, 2013), which, in turn, guides the fragmentation of cDNA (Ma et al., 2017), and the depth and the uniformity of the coverage by sequencing reads (Benjamini and Speed, 2012), are influenced by the GC content. In the following analysis, we aimed at finding out whether any characteristic related to the fragmentation pattern of cfDNA within the locus of interest may influence the depth and the uniformity of coverage with amplification based sequencing reads.

For the ITCHP2 panel, each amplicon was matched to an individual nucleosome map and evaluated according to four features: *(i)* absolute count of experimentally observed continuous cfDNA fragments spanning the whole amplicon (Feature A), *(ii)* read signal amplitude within the amplicon (Feature B), *(iii)* read signal change at the boundaries of amplicon (Feature C), and *(iv)* read signal shape defined as the area between its linear approximation and itself (Feature D) (Figure 5C). Uniformity of the coverage was defined as a coefficient of inter-individual variation in read coverage between all cfDNA samples. To calculate the robustness of the nucleosome mapping, we assessed the inter-sample variance of the defined features calculated for each amplicon. Averaged coefficients of the variation of features D, B and C were at 390, 68, and 38%, respectively, pointing at significant inter-sample variation.

**FIGURE 6:**
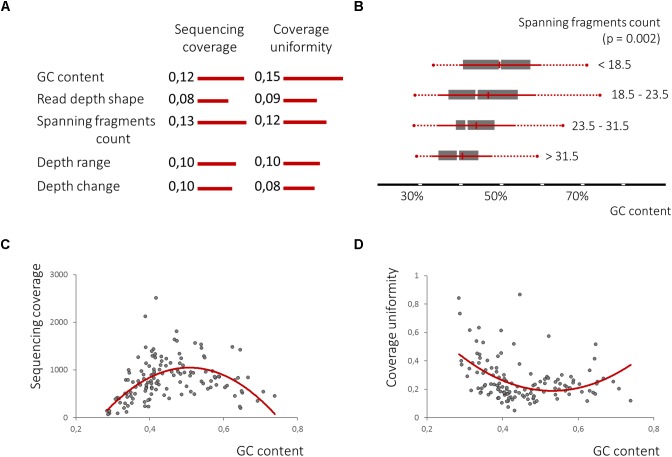
The nucleosome-guided cfDNA fragmentation pattern influences amplicon mean coverage and its uniformity across samples. **(A)**. RReliefF ranking of the defined features in relation to amplicon mean coverage and its uniformity across samples listed in comparison with GC content ranks, previously shown to have strong non-linier correlation with both dependent variables **(C,D)**, demonstrates the significance of all four features for the prediction of target variables, though linear correlation between spanning fragment counts and GC content was observed **(B)**.

Further, we estimated the feature quality, employing the RReliefF method (Robnik-Sikonja and Kononenko, 2003) estimating how well their values distinguish between target variables that are near to each other. Despite previously demonstrated low robustness of the nucleosome associated features, the count of spanning fragments (Feature A) was ranked even higher than the GC content, while the other three features, B, C, and D, closely followed feature A and the GC content (Figure 6A). This finding indicates that uniformity of the locus coverage, with amplified sequencing reads, may depend on the underlying pattern of cfDNA fragmentation.

**FIGURE 7:**
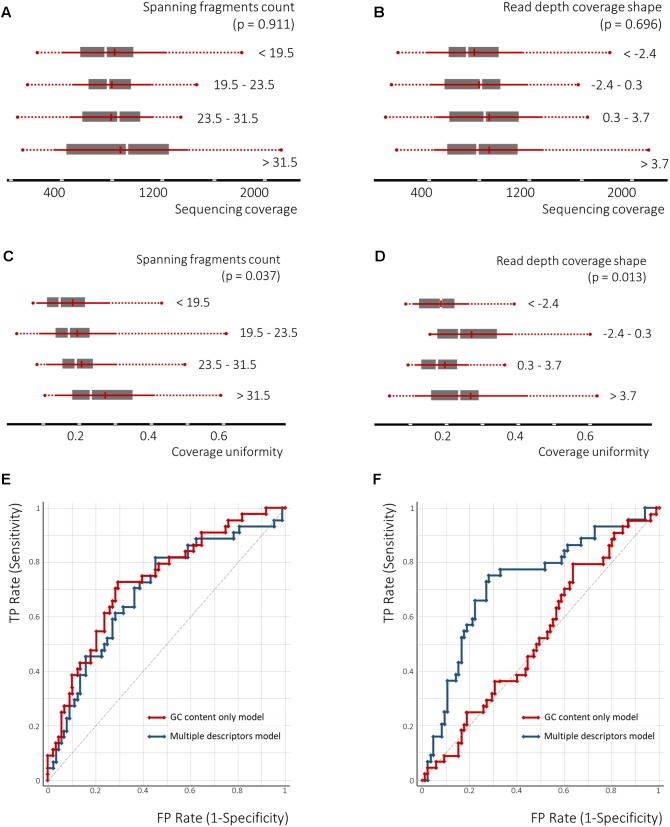
The nucleosome-guided cfDNA fragmentation pattern facilitates the prediction of amplicon coverage uniformity across samples. The ANOVA test demonstrates dependency between spanning fragment counts and coverage uniformity as well as read depth overage shape and coverage uniformity **(C,D)**, though no similar relation to the mean amplicon coverage across the samples was observed **(A,B)**. The SVM classification model utilizing the GC content as a single feature, or in combination with the cfDNA fragmentation pattern defining features, demonstrates the significance of the latter to predict amplicon coverage uniformity across samples **(F)**, but not of the mean sequencing coverage across samples **(E)**.

Univariate polynomial regression of the sequencing coverage depth and its coefficient of variation based on the GC content with second degree polynomial yielded coefficients of determination of 0.29 and 0.19, respectively. Furthermore, GC content equal-frequency discretization (four groups) and analysis of variance of both dependent variables between groups, yielded a *p*-value of less than 1e-6. Thus, a strong non-linear correlation between the GC content, a sequencing coverage and its uniformity (Figure 6C,D) was detected. Despite significant linear correlation between counts of spanning fragments and the GC contents (Figure 6B), no similar relationship between this feature and sequencing coverage was seen (Figure 7A,B). In contrast, as for coverage uniformity, both spanning fragments, count and read depth coverage, shape the demonstrated correlation in relation to it (ANOVA test *p*-value of 0.037 and 0.013, respectively) (Figure 7C,D). No correlation was seen for depth change or depth range (data not shown).

Finally, we tested the performance of the SVM classifier for its prediction of coverage depth and coverage uniformity by either employing the GC content as a single feature or in a combination with all the other features analyzed above. Following 3-groups equal-frequency discretization, the target classes were defined as coverage depth in the lowest third tertile and coverage uniformity in the highest third tertile. For predicting the depth of coverage, GC content in combination with depth change (Feature C) were selected as features. To predict the uniformity of coverage, GC content in combination with the spanning fragment counts (Feature A) and read depth shape (Feature D) were selected as features. A radial basis function (RBF)-kernel utilizing SVM classifier was then applied, using threefold cross-validation. Performance of the SVM classifiers, built upon several features for predicting coverage uniformity, was better than that of the GC-content only classifiers (areas under the receiver operating curve (AUROCs) of 0.75, 95% CI: 0.750–0.752 vs. 0.65, 95% CI: 0.63–0.67; precision – 0.74 vs. 0.68). This indicates that non-GC content features may aid in the prediction of the amplicons with a high coverage variation across samples. For coverage depths, however, applying a similar strategy has not resulted in a significant improvement (AUROCs of 0.69, 95% CI: 0.68–0.70 vs. 0.70, 95% CI: 0.70–0.71; precision – 0.69 vs. 0.69) (Figure 7E,F).

## Discussion

The share of cfDNA fragments originating from tumor rather than normal tissues, may vary greatly among patients. In early-stage disease, the share could be as low as 0.01% of the total cfDNA (Thierry et al., 2017). Because of that, the issue of the detection of low frequency mutant alleles, represents one of the biggest technical challenges to the development of diagnostic and prognostic assays involving the sequencing of cfDNA. In this study we examined various approaches to increase diagnostic and analytical sensitivity of the detection of somatic mutations in liquid biopsy samples.

In a heterogeneous cohort of patients, the liquid biopsy was performed at baseline, at disease progression and/or within the framework of disease monitoring. Overall diagnostic sensitivity of NGS to detect EGFR mutations in cfDNA was at 83%. Of note, when we limited the sample set to the plasma specimens with DNA concentration of 20 ng/ml and higher, the false negative rate was reduced from 17 to 0%. This observation points at low concentrations of cfDNA samples as a primary contributor to imperfect sensitivity of the liquid biopsy assays and at a necessity to either improve the recovery of tumor DNA fragments, or to require cfDNA profiling labs to introduce more stringent QC metrics, which may render many samples ineligible for downstream processing.

Sensitivity of cfDNA based mutation detection assays may be aided by an improvement of amplification efficiency. Plasma cfDNA is known to be highly fragmented (Fleischhacker et al., 2011; Klevebring et al., 2014; Figure 2B). Therefore, it is commonly recognized that an increase in length of PCR amplicons may result in the elimination of a majority of the extracted DNA fragments as possible templates. In this study we sought to dissect how much of the amplicon length influences the sensitivity of subsequent mutation detection. For this we performed, to the best of our knowledge, the first comparison of two amplicon based NGS panels characterized by a substantial difference in average amplicon length (Figure 2A). The comparison was performed in relation to the panels’ diagnostic and analytical sensitivity. Surprisingly, the yield of both the germline and somatic mutations between two panels were completely concordant, pointing at an irrelevance of amplicon size of the specified short range to diagnostic sensitivity of resultant assays.

As a particular example defying “the shorter amplicon, the better amplification efficiency” logic, we dissected the detection of EGFR exon 19 deletion alleles by amplicons of 138 and 168 nt in length. Based on the area under the fragment length distribution curves (Figure 2B), mutant alleles should be amplified 1.45 times more efficiently than wild-type ones by the panel with larger amplicons, while the panel with shorter amplicons would be 1.04 times more efficient for mutant cfDNA fragments. Considering that tumor-derived cfDNA fragments are even shorter than normal tissue-derived ones (Jiang et al., 2018), these rates would increase to 1.84 and 1.16, respectively (Figure 3). This should result in approximately and increase of 1.6 times of the mutant allele frequencies detected with a larger-amplicon panel as compared to a smaller-amplicon panel. In our experiment, no statistically significant difference in mutant allele frequencies was noted, with the observed trend being the opposite to what was expected, indicating that the size of the amplicons does not contribute to the analytical sensitivity of cfDNA assays.

Notably, our observations contradict some previous work (Chan et al., 2004; Koide et al., 2005), which show a length-dependent decrease in efficiency of amplification of cfDNA templates in up to a 250 nt fragment range, which corresponds to the mononucleosome fraction representing approximately 85% of all cfDNA fragments (Figure 2B). In these previous studies, the yield of DNA dropped by almost 30 and 60% when using amplicons with a size of 145 nt instead of 105 and 201 nt instead of 145 nt, while for amplicons with larger sizes no pronounced effect was observed. Furthermore, another study demonstrated that increases in the DNA yield may be observed at a lower amplicon size range: a direct digital PCR comparison of the 50 bp to the 84 bp amplicon resulted in significant favoring of the shorter amplicon (Koide et al., 2005; Sikora et al., 2010). It is important, however, to note that reported observations were obtained in course of analysis if cfDNA samples collected either from healthy individuals or in setting of prenatal diagnostics aimed at amplifying fetal cfDNA and, therefore, cannot be directly projected onto the templates of tumor-derived cfDNA which is known for the shorter sizes of its fragments (Pinzani et al., 2011; Mouliere and Rosenfeld, 2015) and lower integrity (Underhill et al., 2016). The studies of cfDNA specimens collected from patients with tumors show that 60 bp fragments are almost five times more abundant than 150 bp ones, thus pointing at the necessity to use amplicons with sizes of 100 bp or lower (Mouliere et al., 2011).

Importantly, in many cases, reaping the benefit of shorter amplicon size may not be possible due to complications arising from the necessity of the precise positioning of the primers restricting optimization of their GC content, matching melting temperatures and preventing oligonucleotide dimerization. While designing PCR systems for select loci may be still possible, with EGFR analysis being the common example (Reckamp et al., 2016), the introduction of ultra-short amplicons into highly multiplexed systems aiming at a broader molecular profiling of human tumors, may not be feasible. Particular concerns about this multiplexing precluding approach to the amplicon design are owed to the recent observations of a wide mutational spectrum in the liquid biopsies of metastatic cancer patients and its relevance to possible inclusion in clinical trials (Rothé et al., 2014; Frenel et al., 2015). In light of an obvious necessity for multiplexing, the finding that varying amplicon sizes in a range from 140 up to 170 nt does not influence analytical sensitivity is significant, as it shifts the attention of panel designers from minimizing the length of the amplicons to optimizing compatibility of oligonucleotides.

Additionally, cfDNA as a template for a designed PCR-based assay may introduce a set of additional restraints. Both the prevalence of cfDNA fragments of certain sizes and the fragmentation patterns depend on the positioning of the nucleosomes within its tissue of origin. To describe this novel complex variable depicting nucleosome positioning, we introduced four features namely, a spanning fragment count, a read depth change, a read depth range and a read depth shape (Figure 5C), which collectively portray the coverage of select amplicon by experimentally obtained WGS reads. When read coverage maps of WGS-sequenced cfDNA fragments from pooled plasma of healthy patients were aligned to the amplicons employed for liquid biopsy analysis of patients with NSCLC, these four features were utilized to determine the extent of the influence of nucleosome positioning on two dependent variables: sequencing coverage and coverage uniformity. A SVM-based classifier demonstrated that combining the GC content with spanning fragment counts and read depth shape, results in an increased accuracy of prediction of both dependent variables. Therefore, this variable should be taken in consideration when designing PCR primer systems.

Nevertheless, the overall robustness of nucleosome positioning remains unclear. It is known that several regulatory events defining the gene expression require the strict positioning of nucleosomes; these events are typically associated with promoter regions (Hesson et al., 2014; Lövkvist et al., 2018). However, nucleosome positioning is not absolute, and even with major shifts in gene expression, some cells fail to change nucleosome configuration (Small et al., 2014), thus, indicating an underlying complexity of nucleosome positioning. Importantly, the majority of clinically relevant mutations are located within exons, which, according to the current view of cfDNA nucleosome maps, do not retain a strict pattern of cfDNA fragmentation. Therefore, nucleosome arranging within such exons may be variable, either between molecular subtypes of the same disease or even between normal tissue specimens. Nevertheless, despite a potential for low robustness, a substantial correlation observed between nucleosome maps revealed by unbiased read coverage in cfDNA from healthy patients, and the sequencing coverage and its uniformity in amplicons obtained in cfDNA of patients with NSCLC, indicates that the efficiency of amplification may be improved if the unbiased read coverages are taken into account.

In conclusion, low plasma cfDNA concentration remains the major factor that limits the sensitivity of liquid biopsy assays. Above we showed that the design of a highly multiplexed system equally tolerates amplicons in the range of 140–170 bp in size, thus allowing the shift of attention toward the melting temperature, GC clamps, cross homology and other controllable variables. We have also provided evidence that the nucleosome placement in the tissue of origin and the resultant genome-wide cfDNA fragmentation pattern, may be used as a guide for primer positioning to improve both the sequencing coverage and its uniformity.

## Data Availability

The datasets generated for this study can be found in the Sequence Read Archive under accession number SRP167082 (https://trace.ncbi.nlm.nih.gov/Traces/sra/?study=SRP167082). The additional datasets analyzed for this study can be found in the Sequence Read Archive under accession number SRP061633.

## Author Contributions

VM, AB, MI, and SM designed the work. PC, KL, and VB collected the samples. ER performed the experiments. All authors participated in the interpretation of the results and in writing the article.

## Conflict of Interest Statement

ER, SM, VM, and AB were employed by company Atlas Oncology Diagnostics, Ltd. The remaining authors declare that the research was conducted in the absence of any commercial or financial relationships that could be construed as a potential conflict of interest. The reviewer, TT declared a past co-authorship with one of the authors AB to the handling Editor.
